# An On-Machine Measuring Apparatus for Dimension and Form Errors of Deep-Hole Parts

**DOI:** 10.3390/s24237847

**Published:** 2024-12-08

**Authors:** Jintao Liang, Xiaotian Song, Kaixin Wang, Xiaolan Han

**Affiliations:** 1 School of Mechano-Electronic Engineering, Xidian University, Xi’an 710126, China; 2 College of Mechanical Engineering, Xi’an Shiyou University, Xi’an 710065, China

**Keywords:** deep hole, on-machine measure, inner diameter, straightness error, roundness error

## Abstract

The precise measurement of inner dimensions and contour accuracy is required for deep-hole parts, particularly during the manufacturing process, to monitor quality and obtain real-time error parameters. However, on-machine measurement is challenging due to the limited inner space of deep holes. This study proposes an automatic on-machine measuring apparatus for assessing inner diameter, straightness, and roundness errors. Based on the axial-section measurement principle, an integrated measuring module was designed, including a self-centering mechanism, a diameter measuring sensor, and a positioning reference sensor, all embedded within a control system. On this basis, calculations of the inner diameter, and evaluations of the straightness and roundness errors are presented. Experimental verification is conducted on a blind deep hole with a nominal 100 mm inner diameter and 700 mm depth. Compared with measurements performed on a coordinate measuring machine (CMM), which is limited to a maximum hole depth of 300 mm, the proposed apparatus achieved full-depth on-machine measurements. Meanwhile, the measurement results were consistent with the data obtained by the CMM. The straightness error is considered less than 0.05 mm, and the roundness error is considered less than 0.015 mm. Ultimately, without requiring any additional reference platform, the proposed apparatus shows promise for measuring deep-hole parts on various machine tools, with diameters of no less than 80 mm and theoretically unlimited hole depth.

## 1. Introduction

Deep-hole parts are defined by a depth–diameter ratio greater than 5 and are widely used in various high-end productions, such as engine vortex shafts, landing buffers, gun barrels, energy pipelines, hydraulic cylinders, etc., [1,2,3]. The manufacturing quality of these parts has a significant influence on assembly accuracy, operation performance, stability, and service life of the entire equipment [4,5]. As the required machining performance increases, the need for higher measurement accuracy also rises. However, it is challenging to obtain dimensional and contour information, especially during on-machine processing of deep-hole parts [6,7]. At present, on-machine measurement of deep-hole parts mainly relies on manual operation using simple tools such as inner diameter gauges and ultrasonic thickness indicators. These methods are limited by low measurement precision and efficiency, as well as a restricted measurable range. In addition, advanced measurement equipment, such as coordinate measuring machines (CMMs) and laser absolute trackers, exhibit high measurement precision but are unsuitable for on-machine measurement due to their high facility costs [8,9].

In addition to hole diameter, hole form errors are crucial machining indexes, including roundness error, straightness error, etc. The roundness error is defined as the minimum distance between two concentric circles containing the actual contour of the same cross-section, reflecting the deviation of the diameter along the circular contour [10]. The straightness error is defined as the minimum distance between two concentric cylinders that contain the connecting line of all center points of the circular cross-sections along the deep hole [11]. Compared to the measurement of outer profiles, measuring hole diameters and form errors is more complex and challenging. As the diameter decreases, it becomes increasingly difficult to position the measuring devices inside the hole. In addition, as the depth–diameter ratio increases, implementing full-depth measurements becomes even more difficult.

Many studies have been conducted to exploit various measuring devices and assessment methods for deep-hole measurement. According to different hole diameters, the measurement sensor should first be determined, which can mainly be classified into contact and non-contact types [12,13]. Contact-type measurements detect displacement or deformation between the tactile probe and the measured contour, converting this into an electrical signal. Non-contact sensors are mainly based on optical measurement methods, such as laser displacement sensors, optical fibers, charge-coupled device (CCD) cameras, interferometers, etc.

For micro holes with a diameter less than 10 mm, micro-scale sensors are required, such as optical fiber sensors [14]. Wang, et al. [15] proposed an ultracompact optical fiber Fabry–Perot interferometer for measuring the inner diameter of capillary tubes, achieving a relative measurement error of less than 2%. In addition, Chang, et al. [16] developed a micro probe integrated with a capacitor and a sensing circuit for micro-hole measurement, with a repeatability error of ±0.78 mm and a linearity error of 1 μm; however, the maximum measuring depth was only 15 mm. Ma et al. [17] designed a pinhole diameter measuring device using a cylindrical capacitive probe, capable of measuring diameters between *ϕ*1.85 mm and *ϕ*7 mm, with a depth limit of less than 18 mm. Elfurjani et al. [18] introduced a rotating wire probe using acoustic emission (AE) for contact measurement, successfully measuring micro holes with diameters of less than 1.0 mm and depths of 10 mm, and reconstructing the 3D contour. He et al. [19] employed a tungsten probe stylus, with a sphere diameter of approximately 60 mm, for vertical insertion into micro holes driven by a self-developed micro CMM. The measurement uncertainties for diameter and roundness error were 192 and 226 nm, respectively. For hole diameters ranging from 10 mm to 40 mm, more probes and sensors can be placed into the hole. Coordinate measuring machines (CMMs) are typical contact-based probes used for these measurements [20], but the measurable hole depth is limited to several hundred millimeters [21]. Bian et al. [22] constructed a hole diameter measuring machine based on spherical scattering electrical-field probing, achieving a sensor resolution of 1 nm, and 0.2 mm uncertainty with 20 mm diameter. Mahammad et al. [23] employed a ruby probe with a 3D length-measuring machine, an autocollimator, and an angular positioning datum to evaluate probing deflection, achieving a diameter measurement uncertainty of up to 140 nm. Zhu et al. [24] presented a three-dimensional inner surface inspection system based on circle-structured light, achieving an absolute error of 3 μm, and a defect resolution of 0.02 mm. Due to the narrow space of micro and small holes, the motions of the sensor or probe are driven by external scanning platforms, which would not be suitable for on-machine measurement. Gerken et al. [5] developed a BTA drilling process-parallel measurement system using three ultrasonic sensors to measure wall thickness and assess straightness error, although the measurable straightness error was limited to less than 0.1 mm, with questionable precision.

By contrast, for hole diameters larger than 40 mm, appropriate mechanisms can be designed for on-machine measurement with sufficient arrangement space. Shi et al. [25] developed a “DN800 diameter-detector” for pipeline detection and 3D reconstruction using multiple sensors, achieving a radial measurement error of less than ±2 mm and a dip angle accuracy of ±0.5°. Jin et al. [26] described an optical non-contact diameter measurement using a disk beam probe; a wide-field lens and image sensor were set up to collect cross-sectional images, with the measurement range depending on the longitudinal magnification of the lens. Katsuki et al. [27] constructed a laser-guided system for small-sized holes with a laser interferometer, CCD, and laser diode; however, the measurement error was large. Pan et al. [28] proposed a novel deep-hole inner surface 3D reconstruction device using a multi-line structured light projection system (MSLPS), for 155 diameter deep-hole measurement, achieving a maximum absolute error of 0.036 mm. Song et al. [29] introduced a multi-sensor integrated measurement system for deep holes with diameters ranging from 150 to 165 mm and lengths from 600 to 20,000 mm, achieving roundness and axis straightness errors of less than 10 μm and a repeatability accuracy of less than 1 μm. Du et al. [30] employed a laser collimation device into the traditional ring beam measurement apparatus to calibrate the ring light point cloud, improving the accuracy of the 3D reconstruction model by 6.287%. Zhao et al. [31] presented a deep-hole measurement instrument based on laser and monocular camera for holes up to 300 mm in diameter, achieving a repeatability error of 0.012 mm using perspective transformation to compensate for sensor thermal errors. It can be seen that with the complex structure and use of multiple sensors for high precision, the measurable minimum diameter would be larger. At present, there is no uniform method to achieve highly efficient, precise, and cost-effective on-machine measurement of the dimensions and contour accuracy of various deep-hole parts.

In recent years, our research group has been committed to the development of equipment for deep-hole measurement. To facilitate on-machine measurement of deep-hole parts with a diameter of at least 40 mm and no theoretical depth limit, a novel measuring apparatus with two contact-type displacement sensors and self-centering mechanism is developed. To eliminate errors from the positioning reference, a self-centering, adjustable-diameter measuring mechanism is designed, which includes an additional displacement sensor for establishing the original reference. Automated measurement with unlimited hole depth feeding is achieved using a miniaturized car and an embedded control system. Subsequently, the least squares method is employed to evaluate the inner diameter, straightness error, and roundness error, respectively. On this basis, an experiment for blind deep-hole measurement is conducted to verify the effectiveness of the proposed apparatus and methodology. The results are compared with measurement data obtained from a CMM to validate the accuracy of the proposed system.

## 2. On-Machine Measuring Apparatus

### 2.1. Measurement Principle

**Figure 1:**
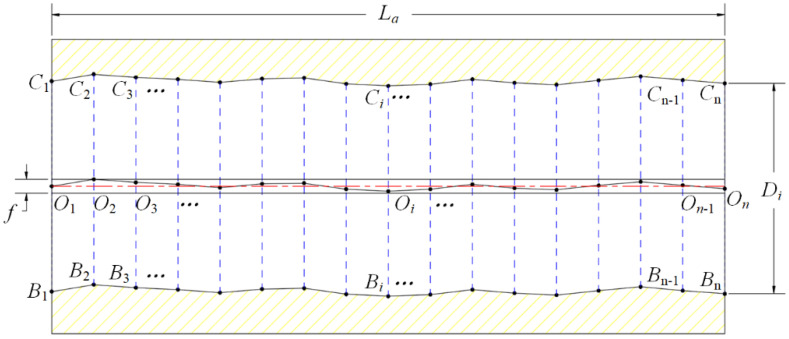
Measurement principle of the axial-section method for deep-hole.

There are two basic principles for deep-hole diameter and form error measurements: cross-section method and axial-section method [32,33]. The cross-section method involves measuring the contour points of a certain circular section, the center-point, diameter, and the roundness error of this cross-section can be evaluated. A series of cross-sections, taken at equal intervals along the deep-hole depth, are used to line up the series of center points in sequence, from which the straightness error can be evaluated. The axial-section method involves measuring the contour points of the upper and lower edge lines in the same axial section of the deep-hole, as shown in Figure 1. At the *i*-th depth circular-section, the diameter *D_i_* and the center-point *O_i_* can be calculated by the two opposite contour-points *C_i_*, *B_i_*, and the straightness error of the deep-hole can be evaluated by the series of center points *O_i_* (*i* = 1, …, *n*). Moreover, the roundness error can be assessed by rotating the deep-hole part and repeating the measurements at certain axial sections.

The main challenge in deep-hole measurement lies in establishing a measurement reference and maintaining consistent reference positioning throughout the whole measurement process. However, the motion of the measuring apparatus contains many positioning errors, especially when there is an increase in the degrees of freedom (DOF). Compared to the cross-section method, the sensor feeding axially is the main motion in the axial-section method, with the deep-hole part rotating only between each axial-section measurement. This makes it easier to ensure inform reference positioning without requiring additional devices. Hence, the axial-section method was employed to develop the on-machine measuring apparatus.

### 2.2. Structure Design

**Figure 2:**
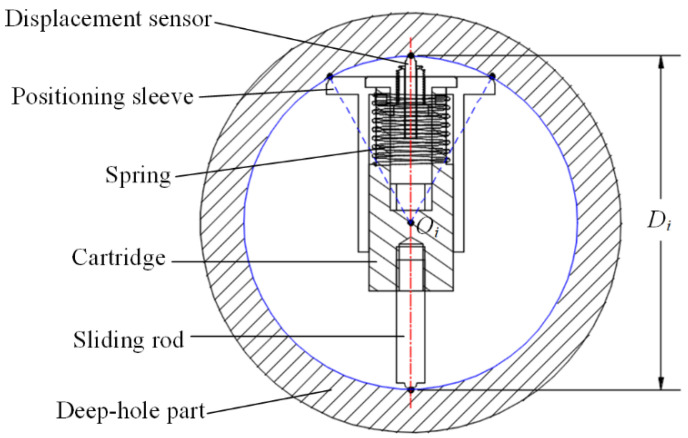
Schematic diagram of three-point centering of circular section.

**Figure 3:**
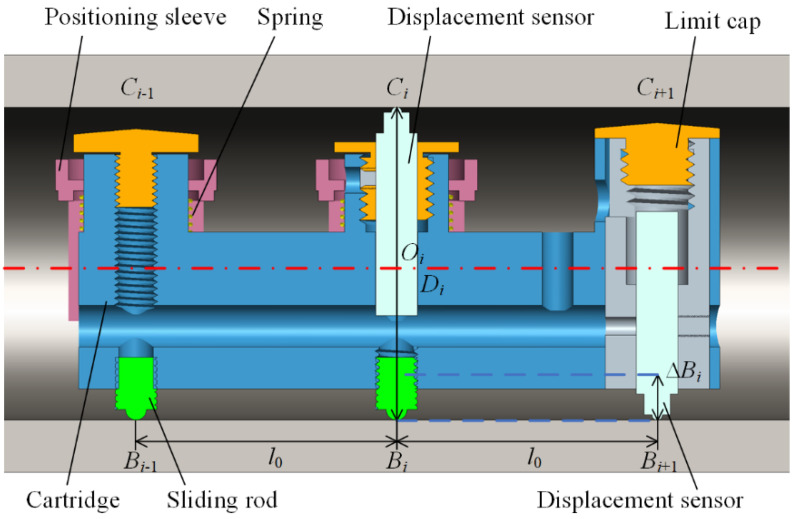
Schematic diagram of the integrated measuring module.

According to the axial-section method, two edge-lines—upper and lower—within the same axial section of the deep hole should be detected. Therefore, the measuring apparatus should feed to arbitrary depth of the deep-hole part to capture the circular section and measure the upper and lower contour points of the edge lines within the same axial section, including the central axis. A three-point centering mechanism is designed, as shown in Figure 2. A sliding rod and a positioning sleeve are mounted on a cartridge coaxially. Two sides of the positioning sleeve are machined as 90° arc-shaped to realize point-contact with the inner contour of the deep-hole. Integrating with the contact-point of the sliding rod, the circular section can be determined by the three contour-points, and the center-point *O_i_* is fixed at the axis of the mechanism. Considering the deviation of the deep-hole, a spring is embedded between the positioning sleeve and the cartridge, so that the positioning sleeve can be adjusted to adapt different circular-section sliding along the deep-hole feeding depth. Self-centering can be realized by this mechanism directly, and the errors generated from the circle fitting and the center-point positioning is avoided. Meanwhile, a displacement sensor is inserted into the cartridge axially opposite to the sliding rod, so that the diameter *D_i_* can be calculated as the distance from the upper point of the sensor to the lower point of the sliding rod. As presented in Figure 2 and Figure 3, the displacement sensor should exhibit compact dimensions and be equipped with an elastic probe. After comparing various options available in the market, a contact pen-type displacement sensor based on the Hall effect was selected. This sensor has a measurement range of 3 mm, a resolution of 0.001 mm, and a repeat accuracy of 0.004 mm.

The three-point centering mechanism can only measure the diameter of the circular contour, but the position deviation among different cross-sections along the deep hole is unknown. The positioning reference should be supplied to establish the uniform global coordinate and then evaluate the form errors. Hence, the integrated measuring module is designed, as shown in Figure 3. Two centering mechanisms are axially arranged apart a certain distance *l*_0_. For high precision and structure simplification, the cartridge is fabricated as a whole part, and one upper displacement sensor is installed axially with the second self-centering mechanism for the diameter measure. With the same distance interval *l*_0_, another lower displacement sensor is arranged to supply a reference positioning.

The measuring process is described as follows: First, the measuring apparatus is placed at the hole port, and then fed as *l*_0_ intervals step by step. At each *i*-th depth position, the diameter *D_i_* (*i* = 1, …, *n*) of the *i*-th circular-section can be measured by the upper displacement sensor. With the next (*i* + 1)-th feeding, the lower contour-point position of the *i*-th circular-section can be measured by the lower displacement sensor. Until the apparatus feeding to the other port or the bottom of the hole, all diameter and lower contour-points position values of all *n* circular-section can be obtained. Employing the positioning value at the port of deep hole as the origin of *x*-*y* coordinate, the center-point of each circular section can be calculated, and then their deviation can be evaluated as the straightness error of the deep-hole. After the apparatus feedback to the starting hole port, with the deep-hole part rotating at a certain angle, the axial-section measurement is repeated to detect two more upper and lower contour points in the same circular section, and the roundness error of each *i*-th circular section can be assessed with all measured contour-points. The detailed calculations concerning the diameter and form errors are discussed in the next section.

**Figure 4:**
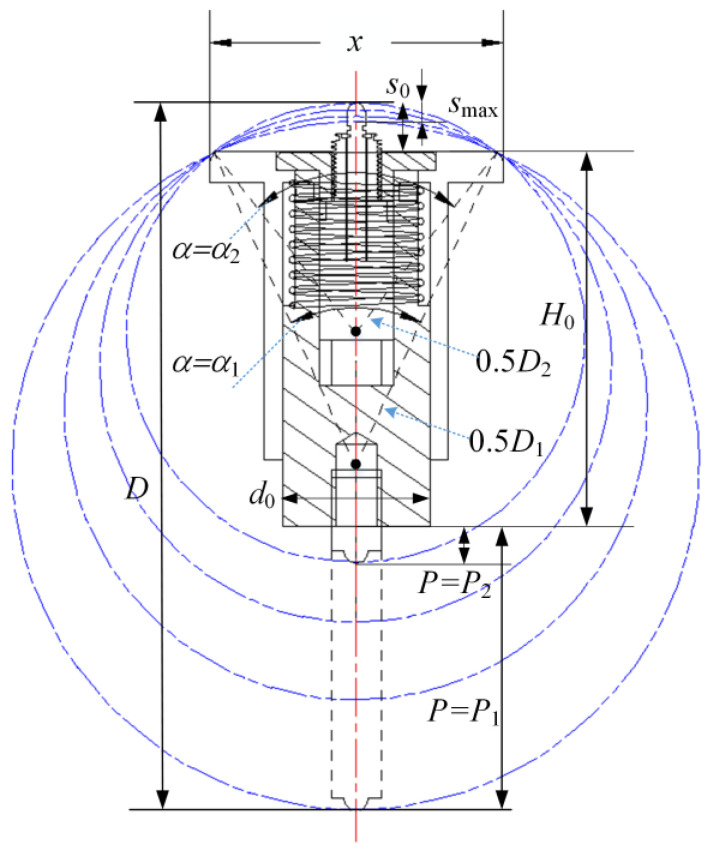
Dimension parameters of the measuring apparatus.

On the other hand, to measure various deep-hole parts with different diameters, the dimension parameters of the apparatus should be calculated first. As shown in Figure 4, with three-point centering, the width of the positioning sleeve *x* and the height of the sliding rod *P* mainly determine the measurable diameter range. Assuming *x* is fixed, with the *P* increases, the central angle *α* between the two contact points decreases, which will increase the positioning error and instability of the three-point centering. A relatively reasonable range of *α* is considered as 50° to 75°, then the measure diameter can be calculated as:

(1) $$D=\frac{x}{\sin\frac{\alpha }{2}}$$

That is to say, a positioning sleeve with a certain width *x* can match a sliding rod with adjustable screw height *P*_1_~*P*_2_, and then the diameter measurable range *D*_1_~*D*_2_ is about 1.64*x*~2.36*x*. In addition, other dimension parameters, such as the diameter *d*_0_ and height *H*_0_ of the cartridge, can be determined quickly.

In theory, for the measuring apparatus, the measurable axial depth of a deep hole is unlimited, whereas the measurable straightness and roundness error are limited by the measure range of the displacement sensor *s*_max_. For a nominal value of diameter *D*, the following equation should be required for maximum measurable straightness and roundness error ± *s*_max_, that is:

(2) $$D=H_{0}+P+s_{0}-0.5\cdot s_{\max}$$

where *P* is the set height of the screw rod, *s*_0_ is the set extending height of the displacement sensor. For instance, the positioning sleeve with *x* = 48 mm is used, that the measurable diameter range is about 80~110 mm. The height of the cartridge *H*_0_ is set to 61 mm. To measure a deep-hole with 100 mm diameter, *P* is set to 32.0 mm, and *s*_0_ is set to 8.5 mm. With the 3 mm displacement sensor, the measurable straightness and roundness errors are about ±1.5 mm, respectively.

### 2.3. Automatic Control System

**Figure 5:**
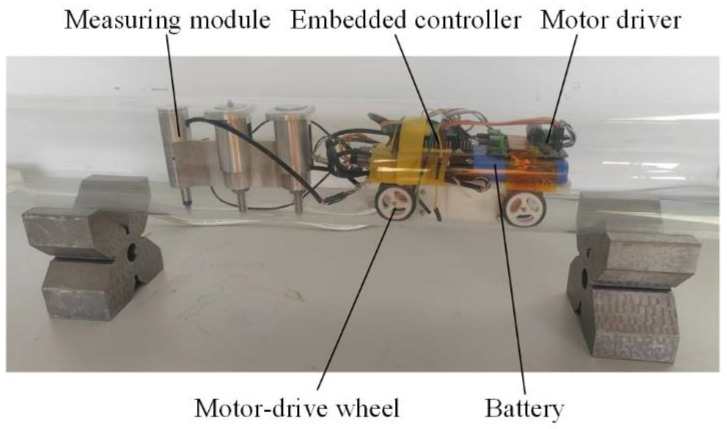
Prototype of the proposed measuring apparatus.

Referring to the above dimension parameters, a prototype is fabricated. For the purpose of on-machine measurement, an automatic measuring process should be integrated with the designed measuring module. As shown in Figure 5, a miniaturized car is designed to drive the measuring module feeding along the deep-hole, the four wheels are directly driven by DC motors. Integrated with Hall angle sensors, the motors are controlled by the motor driver to realize feedback control of feeding position. In addition, the embedded controller with Bluetooth module is employed to receive measurement commands from human machine interface (HMI) of the personal computer (PC), and send back the measurement data to the PC for diameter and form error calculation.

## 3. Diameter and Form Error Calculation

### 3.1. Inner Diameter and Center-Point

According to the above axial-section method and the proposed measuring apparatus, the upper displacement sensor is used to measure the inner diameter of each cross-section *D_i_*, which can be expressed as follows:

(3) $$D_{i}=D_{\max}-\frac{V_{Ci}-0}{V_{\max}}\cdot s_{\max},\;(i=0,1,\cdots n)$$

where *D*_max_ is the maximum distance from the upper point of the sensor to the lower point of the sliding rod, representing the sensor is in the natural state. When the upper sensor is compressed by contacting the contour-point, *V_Ci_* is the output voltage value of the sensor, *V*_max_ is the maximum value of the output value, and *s*_max_ represents the corresponding maximum compressed displacement of the sensor.

An *x*-*y* Cartesian coordinate should be established at the axial-section for positioning reference, as shown in Figure 1. The center-point of the cross-section at the hole port is set as the origin of coordinate *O*_0_ (0, 0), and the corresponding upper point and lower point at the axial-section are *B*_0_ (0, −0.5*D*_0_), *C*_0_ (0, 0.5*D*_0_), respectively.

Then the center-point of the *i*-*th* circular-section *O_i_* (*x_i_*, *y_i_*) can be calculated as follows:

(4) $$\left\{ \begin{array}{l} x_{i}=l_{0}\cdot i\\ y_{i}=\frac{D_{i}-D_{i-1}}{2}+\Delta B_{i}+y_{i-1} \end{array}\right.,\;(i=1,2,\cdots n)$$

where Δ*B_i_* is the displacement variation of the lower points between the adjacent cross-sections, it can be expressed as follows:

(5) $$\Delta B_{i}=\frac{V_{Bi}-V_{B(i-1)}}{V_{\max}}\cdot s_{\max},\;(i=1,2,\cdots n)$$

where *V_Bi_*, *V_B_*_(*i*−1)_ are the output voltage values of the lower displacement sensor at adjacent cross-sections, respectively. The upper point and lower point of the *i*-*th* circular-section *O_i_* (*x_i_*, *y_i_*) can then be obtained, respectively, as: *B_i_* (*x_i_*, *y_i_* − 0.5*D_i_*), *C_i_* (*x_i_*, *y_i_* + 0.5*D_i_*).

### 3.2. Straightness Error Evaluation

Different forms of deviation can occur during deep-hole machining. The straightness error of the hole axis, representing the departure from the actual axis to the ideal reference axis, is the essential technical index to evaluate the hole deviation. Based on the proposed measuring apparatus, the actual axis can be approximated to the line connecting the *n* center-points. In addition, the least squares method (LSM) is employed to determine the reference axis.

In the *x*-*y* coordinate of the axial section, assuming the equation of the reference axis is *y* = *ax* + *b*, which is defined that the sum of squares of the distance from the known center points to the line is minimized. According to LSM, the objective function is established as follows:

(6) $$R_{sl}=\sum_{i=0}^{n-1}{\left(y_{i}-(ax_{i}+b)\right)}^{2}$$

To calculate the coefficients *a* and *b*, the partial derivative of the objective function with respect to *a* and *b* are solved to equal 0, respectively, that is,

(7) $$\left\{ \begin{array}{l} \frac{\partial R_{sl}}{\partial a}=2\sum_{i=0}^{n-1}(y_{i}-(ax_{i}+b))(-x_{i})=0\\ \frac{\partial R_{sl}}{\partial b}=2\sum_{i=0}^{n-1}\left(y_{i}-(ax_{i}+b)\right)=0 \end{array}\right.$$

Then *a* and *b* are obtained, and the least squares fitting line is determined,

(8) $$\left\{ \begin{array}{l} a=\frac{n\cdot \sum_{i=0}^{n-1}x_{i}y_{i}-\sum_{i=0}^{n-1}x_{i}\cdot \sum_{i=0}^{n-1}y_{i}}{n\cdot \sum_{i=0}^{n-1}x_{i}^{2}-\sum_{i=0}^{n-1}x_{i}\cdot \sum_{i=0}^{n-1}x_{i}}\\ b=\frac{\sum_{i=0}^{n-1}x_{i}^{2}\cdot \sum_{i=0}^{n-1}y_{i}-\sum_{i=0}^{n-1}x_{i}y_{i}\cdot \sum_{i=0}^{n-1}x_{i}}{n\cdot \sum_{i=0}^{n-1}x_{i}^{2}-\sum_{i=0}^{n-1}x_{i}\cdot \sum_{i=0}^{n-1}x_{i}} \end{array}\right.$$

The straightness error is evaluated as the maximum distance from the known center-points to the LSM fitting line, multiplied by a factor of two, that is:

(9) $$f_{l}=\max_{i=0}^{n-1}\left(2\cdot \frac{\left|y_{i}-ax_{i}-b\right|}{\sqrt{1+a^{2}}}\right)$$

It should be noted that this result represents the error value for a certain axial section. After rotating the deep-hole part through multiple axial-sections at different angles, the maximum value of *f_l_* is regarded as the straightness error of the deep-hole part.

### 3.3. Roundness Error Evaluation

Using different axial sections taken at various angles, different contour points on the same circular section are obtained, which allows the roundness error to be evaluated. To simplify the measurement process on-machine, different angles are achieved by rotating the angle of the deep-hole part manually. Precise angle positioning of the contour point is not necessary for roundness assessment.

For each circular cross-section, the reference center point can be set to the origin of each local y-z coordinate individually. The fitting diameter of the *i*-th cross-section can be expressed as:

(10) $${D_{i}}^{\prime }=\frac{1}{m}\sum_{j=0}^{m-1}D_{i}^{j}$$

where *m* is the number of the axial-section for two contour points including in once axial measure, so *m* = 180/*θ* times measurement with equal angle interval *θ* is required. *D_i_^j^* represents the diameter of the *i*-th cross-section obtained in the *j*-th axial measurement. The maximum difference of the diameter values among different cross-section is employed to evaluate the roundness error, that is:

(11) $$f_{o}=\frac{1}{2}\max_{i=0}^{n-1}\left(\max_{j=0}^{m-1}D_{i}^{j}-\min_{j=0}^{m-1}D_{i}^{j}\right)$$

It can be seen that the roundness assessment is simple and convenient.

## 4. Experiment Study

### 4.1. Parameter Calibration

**Figure 6:**
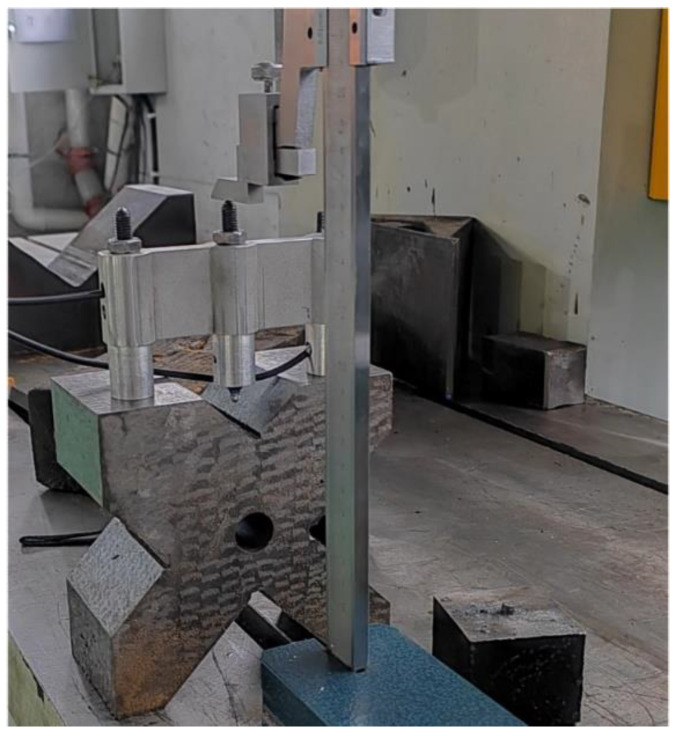
Parameter calibration for the measuring apparatus.

An experimental study is conducted for validation of the proposed measuring apparatus. To ensure measurement precision and consistency, the dimensions of the apparatus, as shown in Figure 2, and the measurement parameters of the displacement sensor, should be calibrated before the measuring process. As shown in Figure 6, based on the reference platform, the height of the two sliding rods is adjusted to coincident by the vernier height gauge, the resolution of which is up to 0.001 mm. The upper displacement sensor is then installed, and the maximum diameter that can be measured is set to 102 mm. Moreover, the maximum compressed distance *s*_max_ is detected as 3.25 mm, and the corresponding output voltage *V*_max_ is detected as 6.37 V.

### 4.2. Measuring Process and Results

**Figure 7:**
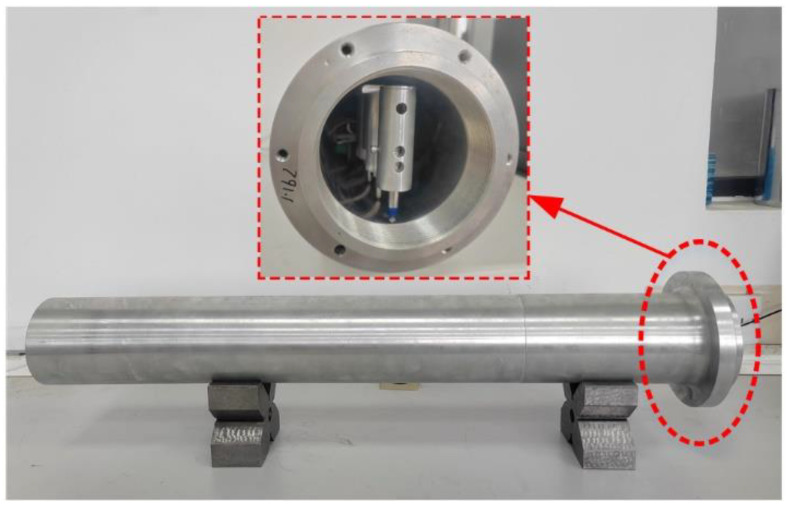
Blind hole measurement achieved by the proposed measuring apparatus.

A blind deep-hole part with 100 mm nominal diameter and 700 mm nominal depth is measured, as shown in Figure 7. In the beginning, the measuring apparatus is placed at the hole port and fed along the hole step-by-step at 50-mm intervals to collect the sensor data automatically, until reaching the hole bottom and then returning to the port. Rotating the part with 60° interval degree roughly to repeat the measuring process, and three-times measurement is carried out.

The feeding time between 50 mm interval is set to 5 s, and the standing time of each position is set to 2 s for sensor data acquisition. For the test piece with a depth of 700 mm, a total of 13 axial position points were detected in one axial-section measurement, and 3 times axial measurement were conducted. Accounting for the apparatus return time and hand operation time, the entire measurement process for the test piece is about 7 min. In further optimization, the feeding time and the standing time can be reduced if less time of on-machine measurement is necessary.

**Figure 8:**
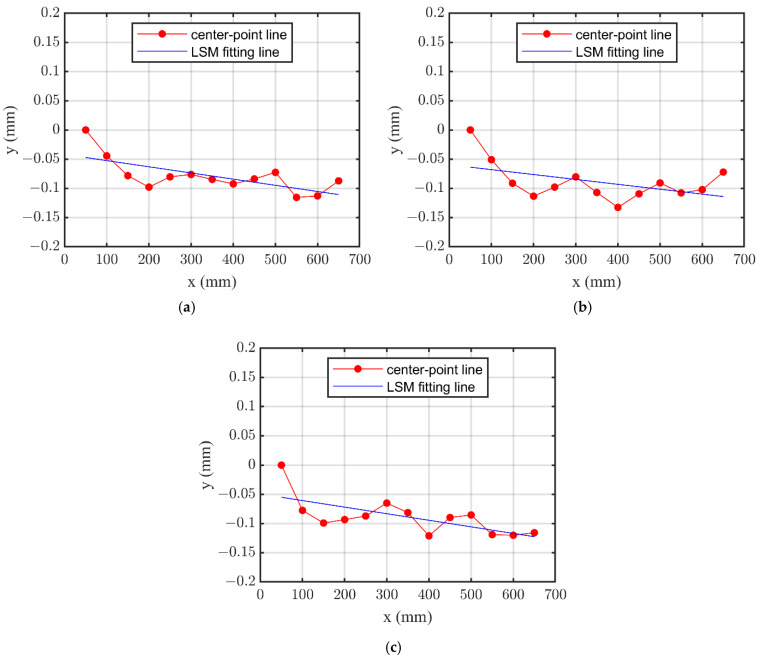
Straightness of the center axis with different direction axial sections. (**a**) 0°-direction axial-section; (**b**) 60°-direction axial-section; (**c**) 120°-direction axial-section.

As shown in Table 1, the original data of output voltage of the sensors (*V_Bi_*, *V_Ci_*) are captured. Through the proposed calculation method to process each axial-measurement individually, the diameter and center-point are obtained. The reference line of LSM is then determined, and the straightness is evaluated, as shown in Figure 8.

The straightness errors of the center axes are 0.0945 mm, 0.1276 mm, 0.1102 mm, respectively, evaluated in the three different axial-sections. The results are approximate, and the waveforms of the center-point line are similar. The maximum value is regarded as the straightness error of the deep-hole part, representing a hole deviation that is coincident with the corresponding angle-direction of the axial section.

To confirm the effectiveness of the result, it is necessary to repeat the whole measurement multiple times on the same deep-hole part with random initial angle-direction that the apparatus placed into the hole port. Therefore, the measurement was conducted three times. Apart from Table 1, the acquired sensor data of another two-times measurement are attached in the Appendix A. The corresponding results of straightness errors are compared, as shown in Table 2. The maximum difference of the three directions are 0.0076 mm, 0.0342 mm, 0.0227 mm, respectively, which indicates that the measure results exhibit favorable consistency, so that the evaluation method of straightness error is feasible.

With the diameter values obtained by different axial-direction measurement, the roundness errors of the series of cross-sections are calculated, respectively, through the proposed method. In addition, the results are compared among the three-times measurement, as shown in Table 3. In each cross-section, the roundness errors evaluated by three-times measurement are basically approximate, the maximum difference among the overall sections is 0.0122 mm. Furthermore, the maximum value of roundness error of the deep-hole part exists at the cross-section of 350 mm depth, the three-times measure results are 0.0587 mm, 0.0559 mm, 0.0538 mm, respectively, which the difference is less than 0.005 mm. Actually, the measurement times conducted is more than three-times, but the difference is not over the maximum value in Table 3. Therefore, only three-times measure results contained, and the repeatability is acceptable.

Although the measurement was conducted in a laboratory setting, no other equipment is needed except the proposed apparatus and a monitor computer. Therefore, on-machine measurement can be realized in the manufacturing workshop, while the part is being processed on the machine tool.

### 4.3. Comparison with CMM

**Figure 9:**
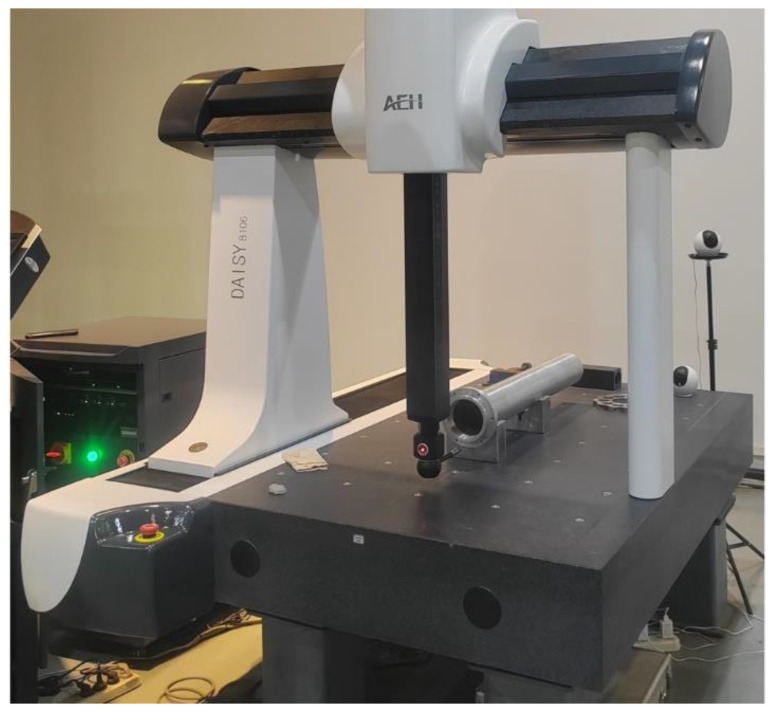
Blind hole measurement achieved by a CMM.

CMM is a precise measurement instrument, whereas it is only suitable for laboratory environment. To verify the precision of the proposed on-machine measuring apparatus, a CMM with 800 mm × 1000 mm × 600 mm measure range and 0.0025 mm probe resolution is employed, as shown in Figure 9. Although the measurement range is enough to arrange the blind deep-hole part, an extension rod is required to feed the probe into the hole, the maximum length of the extension rod is 300 mm. That is to say, only about 300 mm axial depth can be measured from the hole port.

The measurement results between the proposed apparatus and the CMM are compared, as shown in Table 4, where the diameter and roundness errors at the circular-section with 200 mm depth are indicated. The difference among the diameter results is less than 0.04 mm, and the difference among the roundness error is less than 0.015 mm. Furthermore, the value of straightness error measured by the CMM is lower than the apparatus results, that is because only 300 mm depth range can be detected by the CMM. In summary, the precision of the proposed measuring apparatus is reliable, and the measurement and evaluation method are also feasible and effective.

## 5. Measurement Error Discussion

Under the experiment study, measurement error is analyzed to further improve the performance and precision of the proposed measuring apparatus and the form error evaluation method.

### 5.1. Inner Diameter Error Analysis

Regarding the experiment results, the difference among the diameter measurement results is less than 0.04 mm. The highest precision of the deep-hole machining with 100 mm diameter is about 0.01~0.05 mm, thus the measurement precision is basically sufficient. In order to improve the diameter precision, higher resolution of the displacement sensor and greater precision of parameter calibration are considered in further research.

### 5.2. Straightness Error Analysis

The resolution of the displacement sensor and the precision of parameter calibration also influence the measure precision of the straightness error. The straightness error is evaluated individually in each different-direction axial section, and the maximum value is determined as the straightness deviation of the deep hole. Regarding the experiment results, the straightness evaluation error is considered less than 0.05 mm. In theory, increasing the number of axial measurements improves straightness measurement precision; however, the process time consumed is extended. Further analysis is needed to understand the effect of measurement frequency on precision and time efficiency.

### 5.3. Roundness Error Analysis

Based on the proposed method, the measure precision of the roundness error is based on the diameter measure precision. The measurement precision improves as the number of measurements increases. Regarding the experiment results, the roundness evaluation error is considered less than 0.015 mm, which is sufficiently accurate for deep-hole machining.

In particular, when the measuring process is on-machine implemented, although the machining operation is suspended, surface residual chips, burrs, cooling oil, and electromagnetic interference (EMI) from other equipment in the workshop may substantially affect the measurement accuracy. Therefore, appropriate treatment should be adopted to finish the inner surface and eliminate the EMI.

## 6. Conclusions

An automatic on-machine measuring apparatus has been developed for evaluating the inner diameter, straightness error, and roundness error of deep-hole parts. The apparatus, based on the axial-section measurement principle, incorporates a three-point centering mechanism and two Hall-type displacement sensors, allowing for precise evaluation of diameter and form errors. A blind deep-hole part with a 100 mm diameter and 700 mm depth was measured, and experimental results demonstrated that the diameter measurement error was less than 0.04 mm, the straightness evaluation error was less than 0.05 mm, and the roundness evaluation error was less than 0.015 mm. Multiple measurements indicated acceptable repeatability. Furthermore, validation against measurements obtained using a CMM showed a high degree of coincidence with the proposed apparatus, confirming its effectiveness. Consequently, high precision, wide measurable range, low cost, and intelligent on-machine measurement of dimensions and form errors for deep-hole parts can be achieved using the proposed apparatus.

However, there are some limitations in the current apparatus and experimental setup. First, for the tested blind hole, there are currently no feasible methods to evaluate the measurement uncertainty. In further research, statistical analysis with several calibration parts should be conducted to assess measurement uncertainty and repeatability. Second, the minimum hole diameter that can be measured by the current prototype is 80 mm. Structure optimization could enable the apparatus to measure diameters as small as 40 mm. In addition, a measuring apparatus based on optical fiber sensors is planned for future development to facilitate on-machine measurement of smaller deep holes.

## Figures and Tables

**Table 1:** Original voltage data of the sensors in three different axial-direction measurements.

*i*-th Depth (mm)	0°-Section Voltage (V)		60°-Section Voltage (V)		120°-Section Voltage (V)	
	Low Sensor *V_Bi_*	Upper Sensor *V_Ci_*	Low Sensor *V_Bi_*	Upper Sensor *V_Ci_*	Low Sensor *V_Bi_*	Upper Sensor *V_Ci_*
50	3.164	2.540	3.248	2.511	3.230	2.561
100	3.116	2.429	3.274	2.424	3.218	2.403
150	3.139	2.374	3.314	2.365	3.215	2.359
200	3.118	2.325	3.331	2.330	3.200	2.363
250	3.123	2.362	3.282	2.336	3.130	2.340
300	3.139	2.378	3.323	2.391	3.139	2.388
350	3.168	2.376	3.398	2.376	3.229	2.401
400	3.164	2.359	3.378	2.316	3.203	2.310
450	3.141	2.364	3.321	2.333	3.170	2.355
500	3.205	2.418	3.350	2.384	3.233	2.395
550	3.245	2.354	3.343	2.347	3.263	2.344
600	3.217	2.345	3.293	2.333	3.271	2.346
650	3.418	2.496	3.441	2.466	3.450	2.444

**Table 2:** Comparison of straightness errors among three-times measurement.

Measure Times	0°-Section Straightness Error (mm)	60°-Section Straightness Error (mm)	120°-Section Straightness Error (mm)
1st measurement	0.0945	0.1276	0.1102
2nd measurement	0.0909	0.1618	0.1022
3rd measurement	0.0985	0.1487	0.0875

**Table 3:** Comparison of roundness errors among three-times measurement.

*i*-th Depth (mm)	1st Measurement Roundness Error (mm)	2nd Measurement Roundness Error (mm)	3rd Measurement Roundness Error (mm)	Maximum Difference (mm)
50	0.0214	0.0306	0.0314	0.01
100	0.0403	0.0426	0.0401	0.0025
150	0.0446	0.0457	0.0375	0.0082
200	0.0543	0.0536	0.0421	0.0122
250	0.0405	0.0362	0.0293	0.0112
300	0.0469	0.0449	0.0421	0.0048
350	0.0587	0.0559	0.0538	0.0049
400	0.0546	0.0523	0.0495	0.0051
450	0.0459	0.0423	0.0416	0.0043
500	0.0370	0.0309	0.0311	0.0061
550	0.0250	0.0211	0.0230	0.0039
600	0.0194	0.0153	0.0166	0.0041
650	0.0082	0.0094	0.0082	0.0012

**Table 4:** Comparison between the proposed apparatus and the CMM measurement.

Measurement Nos.	Diameter (mm) (at 200 mm Depth Section)	Roundness Error (mm) (at 200 mm Depth Section)	Straightness Error (mm)
Apparatus 1st measurement	100.359	0.0543	0.1276
Apparatus 2nd measurement	100.346	0.0536	0.1618
Apparatus 3rd measurement	100.350	0.0421	0.1487
CMM measurement	100.323	0.0414	0.103

## Data Availability

The original contributions presented in the study are included in the article.

## Appendix A

**Table A1:** Original voltage data of the sensors in the 2nd measurement.

*i*-th Depth (mm)	0°-Section Voltage (V)		60°-Section Voltage (V)		120°-Section Voltage (V)	
	Low Sensor *V_Bi_*	Upper Sensor *V_Ci_*	Low Sensor *V_Bi_*	Upper Sensor *V_Ci_*	Low Sensor *V_Bi_*	Upper Sensor *V_Ci_*
50	3.142	2.541	3.25	2.554	3.262	2.52
100	3.109	2.444	3.276	2.42	3.258	2.398
150	3.116	2.4	3.295	2.355	3.245	2.356
200	3.135	2.362	3.345	2.377	3.246	2.36
250	3.142	2.371	3.284	2.293	3.175	2.314
300	3.146	2.354	3.322	2.415	3.186	2.41
350	3.189	2.369	3.408	2.39	3.262	2.39
400	3.158	2.327	3.363	2.289	3.226	2.291
450	3.147	2.377	3.313	2.36	3.184	2.36
500	3.217	2.406	3.338	2.413	3.25	2.425
550	3.25	2.353	3.333	2.337	3.265	2.335
600	3.226	2.33	3.286	2.334	3.278	2.374
650	3.416	2.51	3.426	2.495	3.453	2.499

**Table A2:** Original voltage data of the sensors in the 3rd measurement.

*i*-th Depth (mm)	0°-Section Voltage (V)		60°-Section Voltage (V)		120°-Section Voltage (V)	
	Low Sensor *V_Bi_*	Upper Sensor *V_Ci_*	Low Sensor *V_Bi_*	Upper Sensor *V_Ci_*	Low Sensor *V_Bi_*	Upper Sensor *V_Ci_*
50	3.136	2.542	3.259	2.49	3.233	2.552
100	3.108	2.446	3.265	2.403	3.253	2.398
150	3.117	2.372	3.253	2.335	3.264	2.357
200	3.14	2.37	3.256	2.355	3.305	2.399
250	3.136	2.337	3.18	2.312	3.251	2.318
300	3.139	2.376	3.192	2.414	3.304	2.437
350	3.192	2.4	3.248	2.393	3.403	2.4
400	3.165	2.323	3.21	2.293	3.359	2.297
450	3.15	2.37	3.172	2.359	3.313	2.366
500	3.221	2.413	3.235	2.428	3.343	2.423
550	3.247	2.341	3.262	2.34	3.337	2.347
600	3.228	2.358	3.272	2.364	3.293	2.346
650	3.417	2.498	3.449	2.501	3.431	2.501
